# Cenchris Contortrix

**Published:** 1892-04

**Authors:** Wm. Steinrauf

**Affiliations:** St. Charles, Mo.


### CENCHRIS CONTORTRIX.

WM. STEINRAUF, M. D., ST. CHARLES, MO.

Mrs. T——, aged fifty-six years, experienced a great deal of pain in the right ovarian region for a long time, and is afraid that a tumor is forming there. Her appetite is fair, excretions and secretions of the body all in order. After several apparently well-indicated remedies caused no change in her condition, I happened to think of what I had read concerning the above remedy, and at once began to give it.

Cenchris$^{cm}$. The pains ceased within three days, and have not returned now for over one year. The pains and distress had existed for about six months before she took the one dose of Cenchris.

Mrs. W——, aged thirty-eight years, has severe pains at each menstrual epoch, and a swelling in the right ovarian region now for over one year. The swelling is about the size of a large orange, and very hard. The menses are very profuse, almost to a flooding; she looks tired and worried, and cries much. Has two children, last over three years old. After the use of several well-indicated remedies there was no change.

Cenchris$^{cm}$. Pain less in one week. Menses only lasted four days the next time, where heretofore they had continued six days and longer. During the next three months she took four doses of Cenchris; pain entirely gone. Menses regular; swelling reduced to the size of an egg, soft and movable. As she considers herself well, she refused further medication.

